# Outcomes of Self-Expanding Versus Balloon-Expandable Transcatheter Aortic Valves in Patients With Reduced Left Ventricular Ejection Fraction: A Meta-Analysis of Observational Studies

**DOI:** 10.1016/j.shj.2026.100843

**Published:** 2026-03-30

**Authors:** Qasi Najah, Sara Saleh, Mustafa Zubaidi, Howard C. Herrmann, Luigi Pirelli, Rebecca T. Hahn, Luis Nombela-Franco, Thomas Pilgrim, Josep Rodés-Cabau, Muhammed Elhadi

**Affiliations:** a Faculty of Medicine, Elmergib University, Alkhums, Libya; b Faculty of Medicine, Hashemite University, Zarqa, Jordan; c Wasit University, College of Medicine, Wasit, Iraq; d Perelman School of Medicine at the University of Pennsylvania, Philadelphia, Pennsylvania, USA; e Division of Cardiothoracic Surgery, New York-Presbyterian Hospital, Columbia University Medical Center, New York, New York, USA; f Cardiology Department, NewYork-Presbyterian/Columbia University Medical Center, New York, New York, USA; g Cardiovascular Institute, Hospital Clínico San Carlos, IdISSC, Madrid, Spain; h Department of Cardiology, Inselspital, University of Bern, Bern, Switzerland; i Quebec Heart and Lung Institute, Laval University, Quebec City, Quebec, Canada; j Faculty of Medicine, University of Tripoli, Tripoli, Libya; k College of Medicine, Korea University, Seoul, South Korea

**Keywords:** Aortic stenosis, Balloon-expandable valve, Self-expandable valve, Systolic dysfunction, Transcatheter aortic valve replacement

## Abstract

Patients with reduced left ventricular ejection fraction (LVEF) undergoing transcatheter aortic valve replacement remain a clinically vulnerable group. Although self-expanding valves (SEVs) and balloon-expandable valves are widely used, the optimal choice in patients with LVEF <40% remains uncertain. We aimed to synthesize the available evidence comparing these two valve types in this high-risk population. We conducted a systematic review and meta-analysis of observational studies comparing SEVs and balloon-expandable valves in patients with LVEF <40% undergoing transcatheter aortic valve replacement. Outcomes included changes in LVEF, aortic gradients, mortality, and safety endpoints. Pooled estimates were calculated using random-effects models, and multivariable meta-regression was performed to adjust for study-level confounding. Five studies comprising 5365 patients were included. SEVs were associated with a greater improvement in 1-month LVEF (mean difference, 2.33; 95% confidence interval [CI], 0.83 to 3.83; *p* = 0.01) and lower mean aortic gradients (mean difference, −2.72; 95% CI, −3.51 to −1.93; *p* < 0.01). Procedural mortality (risk ratio [RR], 0.89; 95% CI, 0.26–3.11; *p* = 0.86), 30-day mortality (RR, 1.52; 95% CI, 0.65–3.56; *p* = 0.33), and 1-year mortality (RR, 1.13; 95% CI, 0.69–1.84; *p* = 0.44) were similar. SEVs carried an increased risk of moderate or worse paravalvular leak (RR, 2.52; 95% CI, 1.46–4.36; *p* < 0.01). While SEVs may offer superior early LVEF improvement, they are associated with a higher rate of paravalvular leaks. Current data are observational and insufficient to recommend one valve type over another.

## Highlights

• SEVs yield greater early LVEF improvement than BEVs in low-EF TAVR patients.
• SEVs show significantly lower post-TAVR transvalvular mean gradients.
• SEVs carry a higher risk of moderate or worse paravalvular leak than BEVs.
• Evidence remains observational, underscoring the need for randomized trials.

## Graphical Abstract

**Figure:**
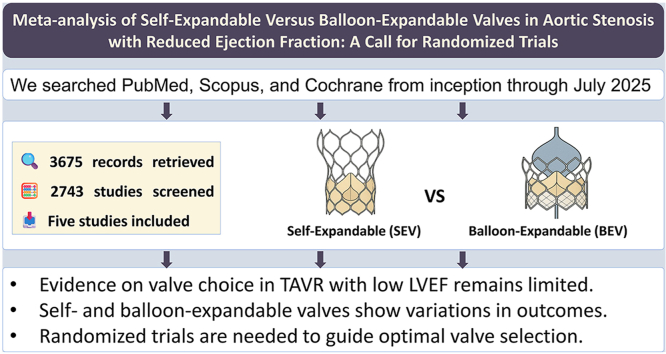

## Introduction

Since its introduction, transcatheter aortic valve replacement (TAVR) has become the preferred therapy for patients with aortic stenosis in its evolution from an option for high-risk surgical candidates to a frontline treatment across all risk profiles.^1^ A pivotal aspect of the TAVR procedure is selecting the appropriate transcatheter heart valve. Traditionally, this choice has been influenced by device availability, operator familiarity, local practice patterns,^2^ and anatomical considerations. However, growing evidence highlights that valve-specific characteristics can meaningfully impact outcomes and should be tailored to individual patient profiles.^3^, ^4^, ^5^

Among available transcatheter heart valves, self-expandable valves (SEVs) and balloon-expandable valves (BEVs) differ in both design and performance. SEVs are often chosen for their favorable hemodynamic properties, including their supra-annular design and lower transvalvular gradients, which may positively impact left ventricular performance.^6^ BEVs, on the other hand, are associated with lower rates of paravalvular leak (PVL) and permanent pacemaker (PPM) implantation in some studies; both have important implications for long-term outcomes.^7^^,^^8^ These differences may be especially relevant in patients with a reduced left ventricular ejection fraction (LVEF). This population is particularly vulnerable to adverse events and is less likely to experience robust recovery after TAVR.^9^

A reduced baseline LVEF (<40%) is a known risk factor for poor post-TAVR outcomes and is a class I indication for aortic valve replacement (AVR), even in asymptomatic patients.^10^ In pooled analyses from the Placement of Aortic Transcatheter Valves (PARTNER) trials, a 5% increase in early post-TAVR LVEF was associated with a 6% reduction in 5-year all-cause mortality. Patients who achieved ≥10-point improvements in LVEF trended toward better survival.^9^ This suggests that even modest improvements in ventricular function carry meaningful prognostic value and that valve-related differences in hemodynamics, conduction, and procedural safety may disproportionately affect this high-risk group.

Although previous randomized controlled trials, such as the CHOICE trial, did not find major differences between valve types in the overall population, subgroup analyses have indicated potential benefits of BEVs in patients with reduced ejection fraction (EF). In CHOICE, patients with LVEF <50% experienced improved composite outcomes with BEVs.^11^ A meta-analysis of 10 randomized trials also demonstrated lower 30-day mortality and PVL rates with BEVs, although it did not specifically examine reduced EF.^4^ Despite these signals, no study to date has directly compared SEVs and BEVs in patients with low LVEF; this leaves a critical evidence gap, which we addressed by conducting a meta-analysis of studies involving patients with reduced LVEF (<40%) undergoing TAVR.

## Materials and Methods

This systematic review was conducted and reported in accordance with the *Preferred Reporting Items for Systematic Reviews and Meta-Analyses* guidelines,^12^ as shown in Supplementary Table 1 in the Supplementary Material. The study was registered in International Prospective Register of Systematic Reviews (PROSPERO) under the identification number CRD420251150878.

### Data Sources and Search Strategy

A comprehensive search of PubMed, Scopus, and the Cochrane Library was performed through July 2025. Search terms included variations of “self-expanding” and “balloon-expandable” transcatheter heart valves and “left ventricular systolic dysfunction” and “transcatheter aortic valve replacement” in the context of aortic stenosis. The complete search strategy is provided in Supplementary Table 2 in the Supplementary Material.

Eligible studies included original studies that directly compared SEVs with BEVs in patients with severe aortic stenosis and a reduced LVEF (<40%). Studies were required to report at least one of the following outcomes: preprocedural and postprocedural LVEF; change in LVEF at 1 year; all-cause mortality; and postprocedural outcomes, including stroke, acute kidney injury (AKI), new PPM implantation, vascular complications, or PVL.

Exclusion criteria included nonoriginal research (e.g., reviews, commentaries), studies without full-text availability, studies not reporting relevant outcomes, pediatric populations, in vitro or animal studies, and gray literature (e.g., abstracts, posters, case reports). No restrictions were placed on language, country, sex, or publication date.

### Data Extraction

A standardized Excel sheet developed for data extraction captured study characteristics, including first author, publication year, country, sample size, design, study period, follow-up duration, and valve models. Baseline population characteristics included mean age, sex distribution, body mass index, baseline LVEF, aortic valve gradient, valve area, Society of Thoracic Surgeons–Predicted Risk of Mortality score, and comorbidities. Clinical outcomes included all-cause mortality at 30 days and 12 months, stroke, AKI, new PPM implantation, length of stay, bleeding, vascular complications, PVL (>mild), change in LVEF, post-TAVR aortic valve gradient, and valve area. The risk of bias was assessed by 2 independent reviewers using the Newcastle-Ottawa Scale. Data were extracted independently by 2 authors and cross-checked by a third for accuracy and consistency.

### Statistical Analysis

All analyses were conducted according to the guidelines in the Cochrane Handbook for Systematic Reviews of Interventions.^13^ We conducted a random-effects meta-analysis using the DerSimonian–Laird estimator for between-study variance (τ^2^) and applied the Hartung–Knapp adjustment to yield more conservative confidence intervals (CIs) under heterogeneity.^14^ Heterogeneity was quantified with Cochran’s Q test and the I^2^ statistic. For dichotomous outcomes (mortality, stroke, bleeding), we pooled risk ratios (RRs) via the Mantel–Haenszel method and reported 95% CIs. Continuous outcomes, including length of stay and post-TAVR LVEF, were synthesized using the generic inverse-variance approach to calculate standardized mean differences. To account for study-level imbalances between treatment groups, a multivariable meta-regression approach was employed. Baseline covariates were selected for adjustment based on three criteria: (1) consistent availability of data across the included trials, (2) evidence of imbalance between the cohorts within the studies, and (3) clinical relevance to the outcomes of interest. The selected covariates were entered as moderators in mixed-effects models. All models were fit in R (v4.3.2) with the meta package (v5.2-0) and metafor package (v4.8-0), and forest plots were generated with ggplot2 (v3.5.2).^15^, ^16^, ^17^

## Results

### Search Results and Characteristics of the Included Studies

**Figure 1:**
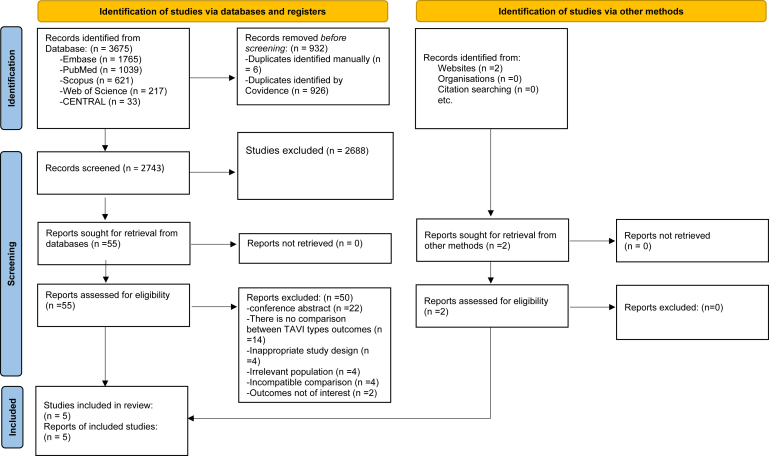
**PRISMA flow diagram showing the process of screening the studies.** Abbreviations: n, number; PRISMA, Preferred Reporting Items for Systematic Reviews and Meta-Analyses; TAVI, transcatheter aortic valve implantation.

Our search included MEDLINE, Embase, Cochrane CENTRAL, and Scopus from inception to July 2025, resulting in the screening of 2743 studies and the inclusion of five nonrandomized studies with a total of 5354 patients^18^, ^19^, ^20^, ^21^, ^22^ (Figure 1). Of these, a total of 1824 patients were identified as having a low EF (<40%)—1041 patients in the SEV group and 783 patients in the BEV group. The average pooled baseline EF was 32%. All five studies were retrospective in design and were conducted across Europe and the United States. Study periods ranged from 2006 to 2025. Valve models were inconsistently reported, with the most common being the Medtronic CoreValve/Evolut series for SEV and the Edwards Sapien series for BEV (Table 1). In Table 2, we provide an overview of the major RCTs comparing SEV and BEV. The risk of bias analysis reveals consistent weaknesses in the comparability domain, suggesting inadequate control for confounding and a cautious interpretation of pooled results (Supplementary Table 3) (***Graphical Abstract***).

**Table 1:** Characteristics of included studies in patients with LV systolic dysfunction undergoing TAVI

Variable	El-Chilali et al. 2020 (Germany)^19^	Matta et al. 2024 (France)^20^	Mustafa et al. 2022 (US)^18^	Nakase et al. 2025 (Switzerland)^21^	Giordano et al. 2023 (Europe)^22^
Study design	Retrospective	Retrospective	Retrospective	Retrospective (PSM)	Retrospective
Study setting	West-German Heart & Vascular Center	Toulouse University Hospital	Northwell Health (4 centers)	Bern TAVI Registry (SwissTAVI)	The Low Systolic function and Transcatheter Aortic Valve Implantation registry
Study period	January 2006–July 2016	January 2016–December 2020	January 2016–December 2020	August 2007–December 2022	Not reported
Study population (total)	679	977	2028	758	923
Definition of LVSD	LVEF ≤40%	LVEF ≤35%	LVEF ≤40%	LVEF <50%	LVEF <35%
Systolic dysfunction – SEV (n)	49	66	146	293	646
Systolic dysfunction – BEV (n)	92	91	189	465	277
Follow-up duration	1 y	∼3.4 y	1 y	5 y	1 y
Primary endpoint	1-y all-cause mortality	Long-term all-cause mortality	Composite outcome	5-y all-cause mortality	Composite outcome
Main conclusion	BEV is associated with better 1-y survival vs. SEV in LVSD.	SEV may offer improved long-term survival vs. BEV.	No clear superiority of SEV vs. BEV at 1 y.	BEV is superior to SEV at 5 y despite SEV's hemodynamic benefit.	TAVR is favorable even in extremely reduced LVEF.
Valve models – SEV	CoreValve, Evolut R (Medtronic)	Not reported	CoreValve (Medtronic)	CoreValve, Evolut R, Evolut PRO, PRO Plus	Unspecified
Valve models – BEV	Cribier-Edwards, Sapien, XT, 3 (Edwards)	Not reported	Sapien (Edwards)	Sapien XT, 3, 3 Ultra (Edwards)	Unspecified

Abbreviations: BEV, balloon-expandable valve; LV, left ventricular; LVEF, left ventricular ejection fraction; LVSD, left ventricular systolic dysfunction; PSM, propensity score matching; SEV, self-expandable valve; TAVI, transcatheter aortic valve implantation; TAVR, transcatheter aortic valve replacement.

**Table 2:** Summary of major RCTs comparing SEV and BEV outcomes in TAVR

Trial name	Trial ID	Timing	Sample size (SEV vs. BEV)	Participants	% low EF	Primary endpoint	Summary of findings
CHOICE^8^	NCT01645202	March 2012–December 2013	120 SEV vs. 121 BEV (241 total)	High-risk severe AS, age >75, EuroSCORE ≥20%, STS ≥10%, or surgical contraindication	≈12.4% overall (15% BEV, 9.6% SEV)	Device success (VARC-defined composite: correct valve position, AVA >1.2 cm^2^, mean gradient <20 mmHg, no moderate/severe regurgitation, single implant)	BEV had higher device success (95.9 vs. 77.5%, *p* < 0.001), less residual AR (4.1 vs. 18.3%, *p* < 0.001), fewer second-valve implants (0.8 vs. 5.8%, *p* = 0.03), and fewer PPIs (17.3 vs. 37.6%, *p* = 0.001). Thirty-day mortality was similar (4.1 vs. 5.1%).
SMART^5^	NCT04722250	April 2021–September 2022; 12-mo follow-up	358 SEV vs. 358 BEV (737 randomized, 716 treated)	Severe AS with small annulus (≤430 mm^2^)	4% with EF <40%	Coprimary: (1) death/stroke/HF rehospitalization (noninferiority), (2) bioprosthetic valve dysfunction (superiority)	Clinical endpoint: SEV noninferior to BEV (9.4 vs. 10.6%). Valve dysfunction was lower with SEV (9.4 vs. 41.6%, *p* < 0.001). SEV had better hemodynamics (gradient 7.7 vs. 15.7 mmHg; EOA 1.99 vs. 1.50 cm^2^). Safety similar.
SCOPE I^11^	NCT03011346	February 2017–February 2019; 30-d endpoint	372 SEV (ACURATE neo) vs. 367 BEV (SAPIEN 3) (739 total)	Symptomatic severe AS, ≥75 y, increased surgical risk	Severely reduced EF (<20%) excluded	Composite of death, stroke, bleeding, vascular complications, coronary obstruction, AKI, rehospitalization, valve dysfunction, regurgitation, stenosis	Primary endpoint higher with SEV (24 vs. 16%); noninferiority not met. BEV superior (*p* = 0.0156), with fewer cases of AKI (1 vs. 3%) and paravalvular leak (3 vs. 9%). Hemodynamics favored SEV (lower gradient, larger EOA). PPI similar.
SOLVE-TAVI^23^	NCT02737150	April 2016–April 2018; 30-d endpoint	219 SEV vs. 219 BEV (438 treated)	Symptomatic severe AS, ≥75 y, high surgical risk (EuroSCORE ≥20% or STS ≥10%)	EF <35% in 5.7% SEV vs. 9.1% BEV	Composite of death, stroke, paravalvular leak, and PPI at 30 d	Composite rates are similar (28.4% SEV vs. 25.9% BEV). Mortality (3.2 vs. 2.3%), stroke (0.5 vs. 4.7%), PVL (3.4 vs. 1.5%), and PPI (23.0 vs. 19.2%). No major differences overall.
LANDMARK^24^^,^^25^	NCT04275726; EudraCT 2020-000137-40	January 2021–December 2023; 30-d endpoint	384 SEV (Myval) vs. 384 BEV (Sapien/Evolut) (768 total)	Real-world severe AS, including bicuspid valves (6–8%) and small annuli (31–33%)	Excluded patients with EF <30%	Composite of death, stroke, bleeding, AKI, vascular complications, regurgitation, PPI (VARC-3)	Myval noninferior (25 vs. 27%, RD –2.3%, *p* < 0.0001). Similar individual outcomes. Myval required fewer post-dilatations (10 vs. 21%) and offered intermediate-sized balloons.
LYTEN^26^	NCT03520101	May 2017–January 2022; 30-d endpoint	49 SEV vs. 49 BEV (98 treated)	Patients with small (≤23 mm) failed surgical aortic bioprostheses	Mean EF ∼58%; no low-EF subgroup reported	Valve hemodynamics at 30 d (residual gradients, mismatch, AR)	SEV had lower mean gradients (15 vs. 23 mmHg, *p* < 0.001), fewer patients with residual gradient >20 mmHg (21 vs. 62%, *p* < 0.001), and a trend toward less mismatch (44 vs. 64%). No AR, deaths, or strokes. Clinical outcomes are rare and similar.

Abbreviations: AKI, acute kidney injury; AR, aortic regurgitation; AS, aortic stenosis; AVA, aortic valve area; BEV, balloon-expandable valve; CHOICE, Comparison of Transcatheter Heart Valves in High‑Risk Patients With Severe Aortic Stenosis; EF, ejection fraction; EOA, effective orifice area; HF, heart failure; LANDMARK, Randomized Non‑inferiority Trial Comparing Myval THV Series With Contemporary THV Series in Severe Native Aortic Stenosis; LYTEN, Comparison of Balloon‑Expandable Edwards Valves and Self‑Expandable Evolut R/PRO Systems for Treatment of Small, Severely Dysfunctional Surgical Aortic Bioprostheses; PPI, permanent pacemaker implantation; PVL, paravalvular leak; RD, risk difference; SCOPE I, Safety and Efficacy of the ACURATE neo Valve Compared With the SAPIEN 3 Valve; SEV, self-expandable valve; SMART, SMall Annuli Randomized To Evolut or SAPIEN Trial; SOLVE‑TAVI, Comparison of Second‑Generation Self‑Expandable Versus Balloon‑Expandable Valves and General Versus Local Anesthesia in Transcatheter Aortic Valve Implantation; STS, Society of Thoracic Surgeons; TAVI, transcatheter aortic valve implantation; TAVR, transcatheter aortic valve replacement; VARC, Valve Academic Research Consortium.

### Hemodynamic Outcomes

**Figure 2:**
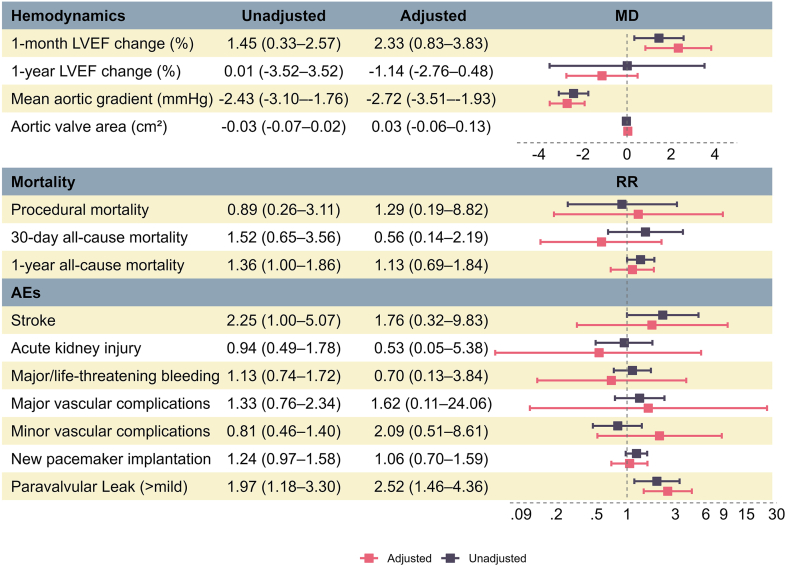
**Forest plot showing the meta-analysis results with and without adjusting for baseline variables.** Abbreviations: AEs, adverse events; LVEF, left ventricular ejection fraction; MD, mean difference; RR, risk ratio.

**Figure 3:**
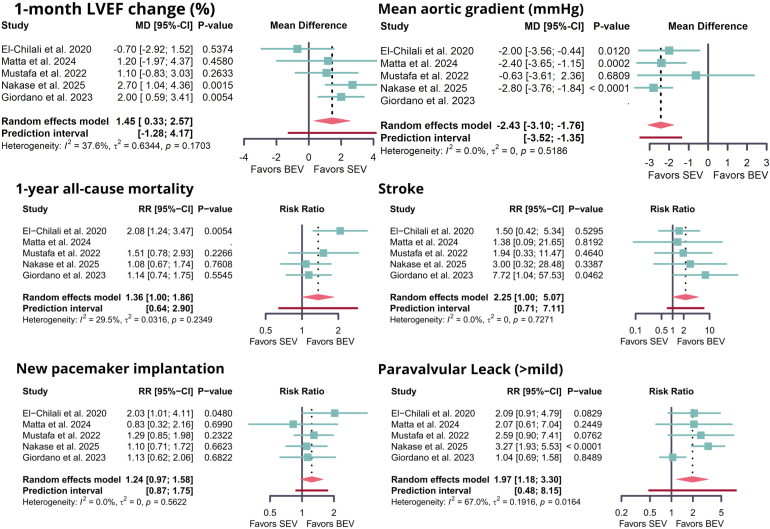
**Forest plot showing the meta-analysis results comparing self-expandable versus balloon-expandable valves.** Abbreviations: BEV, balloon-expandable valve; LVEF, left ventricular ejection fraction; MD, mean difference; RR, risk ratio; SEV, self-expandable valve.

SEVs were associated with lower transvalvular mean gradients (mean difference [MD], −2.43 mmHg; 95% CI, [−3.10 to −1.76]; *p* < 0.01) (Figures 2 and 3) and greater improvements of 1-month LVEF (MD, 1.45; 95% CI, [0.33–2.57]; *p* < 0.01) (Figures 2 and 3), and the change in LVEF was similar between the groups at 1 year (MD, 0.01; 95% CI, [−3.52–3.53]; *p* = 0.85) (Figure 2 and Supplementary Figure 1). The mean postprocedure aortic valve area was comparable between both valve types (MD, −0.03 cm^2^; 95% CI, [−0.07–0.02]; *p* = 0.57) (Figure 2 and Supplementary Figure 2).

After adjustment for study-level covariates, these findings were consistent: SEVs showed greater improvements in 1-month LVEF (MD, 2.33; 95% CI, [0.83–3.83]; *p* < 0.01) and lower mean gradients (MD, −2.72; 95% CI, [−3.51 to −1.93]; *p* < 0.01). The adjusted analysis also showed no difference in postprocedural aortic valve area (MD, 0.03; 95% CI, [−0.07–0.13]; *p* = 0.50).

### Mortality and Safety Outcomes

No difference was found in procedural mortality between SEVs and BEVs (RR, 0.89; 95% CI, [0.26–3.11]; *p* = 0.86) (Figure 2 and Supplementary Figure 4). At 30 days, all-cause mortality was also comparable (RR, 1.52; 95% CI, [0.65–3.56]; *p* = 0.33) (Figure 2 and Supplementary Figure 3). At 1 year, however, SEVs were associated with higher all-cause mortality (RR, 1.36; 95% CI, [1.00–1.86]; *p* = 0.049) (Figures 2 and 3). The adjusted estimates showed broadly similar results but without apparent differences in procedural mortality (RR, 1.29; 95% CI, [0.19–8.82]; *p* = 0.80), 30-day mortality (RR, 0.56; 95% CI, [0.14–2.19]; *p* = 0.40), and 1-year mortality (RR, 1.13; 95% CI, [0.69–1.84]; *p* = 0.62).

Stroke risk appeared higher with SEVs in crude estimates (RR, 2.25; 95% CI, [1.00–5.07]; *p* = 0.05) (Figures 2 and 3), but this was not reproduced in the adjusted analysis (RR, 1.76; 95% CI, [0.32–9.83]; *p* = 0.52).

No differences were observed in AKI (crude RR, 0.94; 95% CI, [0.49–1.78]; *p* = 0.84; adjusted RR, 0.53; 95% CI, [0.05–5.38]; *p* = 0.60) (Figure 2 and Supplementary Figure 5), major or life-threatening bleeding (crude RR, 1.13; 95% CI, [0.74–1.72]; *p* = 0.57; adjusted RR, 0.70; 95% CI, [0.13–3.84]; *p* = 0.68) (Figure 2 and Supplementary Figure 6), or major vascular complications (crude RR, 1.33; 95% CI, [0.76–2.34]; *p* = 0.31; adjusted RR, 1.62; 95% CI, [0.11–24.06]; *p* = 0.73) (Figure 2 and Supplementary Figure 7). Minor vascular complications were also similar in the crude analysis (RR, 0.80; 95% CI, [0.46–1.40]; *p* = 0.44), with the adjusted model showing a wide, imprecise interval (RR, 2.09; 95% CI, [0.51–8.61]; *p* = 0.31) (Figure 2 and Supplementary Figure 8).

Regarding PPM implantation, SEVs showed a trend toward a higher rate compared with BEVs (crude RR, 1.24; 95% CI, [0.97–1.58]; *p* = 0.09), but this was attenuated in adjusted analysis (RR, 1.06; 95% CI, [0.70–1.59]; *p* = 0.79) (Figures 2 and 3). By contrast, the risk of more-than-mild PVL was consistently higher in SEVs across both crude (RR, 1.97; 95% CI, [1.18–3.30]; *p* < 0.01) and adjusted estimates (RR, 2.52; 95% CI, [1.46–4.36]; *p* < 0.01) (Figures 2 and 3).

### Predictors of Hemodynamic and Clinical Outcomes

Female sex was associated with less improvement in LVEF (−0.07; 95% CI, [−0.11 to −0.03]) and a higher risk of major vascular complications (RR, 2.00; 95% CI, [1.16–3.45]). Lower baseline LVEF was associated with smaller gains in post-TAVR LVEF (−0.35; 95% CI, [−0.59 to −0.10]) and lower postprocedural gradients (−0.27; 95% CI, [−0.51 to −0.02]). Higher PPM implantation rates consistently predicted worse outcomes, including increased 30-day mortality (RR, 1.08; 95% CI, [1.01–1.16]), 1-year mortality (RR, 1.06; 95% CI, [1.02–1.10]), and stroke (RR, 1.13; 95% CI, [1.01–1.26]) (Supplementary Table 3).

## Discussion

We conducted a comprehensive literature review to assess the effectiveness and safety of SEVs compared to BEVs in patients with reduced EF. The lack of randomized controlled trials directly addressing this question leaves the evidence base for this high-risk subgroup extremely limited. Observational studies suggest that SEVs may have higher rates of PVL but have favorable hemodynamic outcomes and greater improvement in left ventricular (LV) function.

The observed hemodynamic benefits of SEVs are consistent with their supra-annular design, which provides a large effective orifice area and lower transvalvular gradients.^27^^,^^28^ These features may be particularly advantageous in patients with systolic dysfunction, in whom reducing afterload and improving forward stroke volume are critical for myocardial recovery. Recent studies, however, have suggested that differences in valve construction result in differences in measured gradients; the BEV is associated with higher echocardiographic and lower invasive gradients than the SEV.^29^ The benefits of echocardiographically lower gradients were tempered by higher rates of PVL with SEVs, a complication of particular concern in patients with reduced LVEF.^30^^,^^31^

Conduction disturbances and the need for a PPM are additional important considerations in this population. Our analysis revealed a trend toward higher rates of PPM implantation with SEVs, although this difference was not statistically significant. This finding spotlights an ongoing debate regarding PPM type selection after TAVR. Importantly, no randomized clinical trials have yet been published that directly compare pacing strategies in this population. Although many patients experience some improvement in ventricular function, chronic right ventricular pacing may induce ventricular dyssynchrony and pacing-induced cardiomyopathy, which may be particularly concerning for patients with systolic dysfunction.^32^^,^^33^

Interestingly, we found that female sex was associated with less LVEF improvement and a higher risk for vascular events. Although our analysis is underpowered, evidence from recent meta-analysis suggests that females are more likely to have vascular bleeding compared to men; this finding needs further research on sex-related anatomic variation that may contribute to a higher risk of bleeding outcomes.^34^

Sex-specific differences in outcomes after TAVR have been noted in prior studies, with female patients often demonstrating higher rates of vascular complications and, in some cohorts, less pronounced LV recovery. Although our analysis was underpowered to explore these associations in depth, the observed trends highlight the need for further investigation into sex-related factors that may influence recovery and procedural risk.

The interpretation of our findings must take into account the potential for selection bias inherent in observational studies. Operators may have been more likely to select SEVs for patients with smaller annuli, which are more common among women. Indeed, a higher proportion of females was observed in the SEV groups of some of the included studies.^18^^,^^19^ Prior studies demonstrating that SEVs have larger effective orifice areas than BEVs^5^^,^^8^ support the hemodynamic advantages we observed. Furthermore, we must acknowledge the potential impact of older-generation valves, as advances in valve technology, implantation techniques, and patient selection over time may introduce temporal heterogeneity that influences outcomes.

Baseline imbalances further complicate the interpretation of comparative outcomes. In Mustafa et al.,^18^ patients treated with SEVs had significantly lower baseline LVEF and higher rates of New York Heart Association class III/IV heart failure; this group may have had a higher burden of disease at baseline. In contrast, El-Chilali et al.^19^ reported a greater prevalence of coronary artery disease and percutaneous coronary intervention in the BEV group. To address these imbalances, we conducted a multivariable meta-analysis, adjusting for study-level covariates. After adjustment, the higher mortality and stroke risk associated with SEVs were no longer significant, while the hemodynamic advantages of SEVs (including greater LVEF improvement and lower mean gradients) remained consistent. However, we must note that these adjustments were based on study-level covariates and therefore cannot account for patient-level confounding. Furthermore, the small number of studies may increase the overfitting in this context.

Most published randomized trials comparing SEVs and BEVs did not enroll patients with significant systolic dysfunction or account for the power to detect differences in this population. For example, CHOICE included only ∼12% of patients with EF < 35%, SCOPE I excluded patients with EF < 20%, and SMART enrolled fewer than 4% with EF < 40%. Consequently, no RCT has been designed to address outcomes in this low-EF population. However, the ongoing BEST trial (NCT05454150) may help bridge this gap. This trial enrolled approximately 2000 low-to-intermediate-risk participants and compared Edwards SAPIEN 3 Ultra RESILIA (BEV) with Medtronic Evolut FX/PRO+ (SEV). Importantly, unlike prior trials, BEST does not impose an EF cutoff. Its 1-year composite outcomes are expected in 2026 and may provide critical evidence to inform valve selection in this vulnerable population.^35^

In conclusion, our study contributes to the limited evidence by systematically analyzing outcomes of SEVs versus BEVs in patients with reduced LVEF and underscoring the urgent need for randomized controlled trials specifically designed to compare valve types in patients with low EF. Until such evidence is available, valve selection should be individualized and incorporate careful consideration of the hemodynamic benefits of SEVs against the risks of PVL and conduction disturbances. Future research should focus on patient-level meta-analyses and RCTs with core laboratory adjudication to support decision-making and optimize TAVR outcomes in patients with reduced EF.

## CRediT Authorship Contributions

Conceptualization: Muhammed Elhadi, Qasi Najah. Methodology: Muhammed Elhadi, Qasi Najah, Sara Saleh, Mustafa Zubaidi. Data curation: Sara Saleh, Mustafa Zubaidi, Qasi Najah. Formal analysis/Statistical analysis: Qasi Najah. Investigation (screening, data extraction): Qasi Najah, Sara Saleh, Mustafa Zubaidi. Visualization (figures, graphical abstract): Qasi Najah, Muhammed Elhadi. Writing – original draft preparation: Muhammed Elhadi, Qasi Najah. Writing – review & editing: Howard C. Herrmann, Luigi Pirelli, Rebecca T. Hahn, Luis Nombela-Franco, Thomas Pilgrim, Josep Rodés-Cabau, Muhammed Elhadi, Qasi Najah. Supervision: Howard C. Herrmann, Luigi Pirelli, Rebecca T. Hahn, Luis, Muhammed Elhadi. Project administration: Muhammed Elhadi. Critical revision of the manuscript for important intellectual content: All authors. Approval of the final manuscript: All authors.

## Data Availability

The data sets generated and/or analyzed during the current study are available from the corresponding author upon reasonable request.

## Ethics Statement

This study is a systematic review and meta-analysis of previously published data and did not require institutional review board approval.

## Funding

The authors have no funding to report.

## Review Statement

The review of this manuscript was managed by Guest Editor Harold Dauerman, MD.

## Disclosure Statement

H.C. Herrmann reports institutional research funding from 10.13039/100000046Abbott, 10.13039/100006520Edwards Lifesciences, 10.13039/100004331Johnson & Johnson, and 10.13039/100004374Medtronic; consultant fees from Affluent Medical, Artedrone, Caranx, Johnson & Johnson, Medtronic, Massachusetts Medical Society, and Microinterventional Devices; and equity holdings in Affluent Medical, Artedrone, Caranx, and Microinterventional Devices. T. Pilgrim reports research grants from the 10.13039/501100001711Swiss National Science Foundation, the 10.13039/501100004362Swiss Heart Foundation, the 10.13039/501100015594Swiss Polar Institute, the Bangerter-Rhyner Foundation, the Mach-Gaensslen Foundation, and the Monsol Foundation. Research, travel, or educational grants were provided to the institution without personal remuneration from 10.13039/501100005035Biotronik, 10.13039/100008497Boston Scientific, 10.13039/100006520Edwards Lifesciences, and ATSens. The institution received speaker and consultancy fees from Biotronik, 10.13039/100008497Boston Scientific, 10.13039/100006520Edwards Lifesciences, 10.13039/100000046Abbott, 10.13039/100004374Medtronic, Biosensors, and Highlife.

The other authors had no conflicts to declare.
