# Supplementary material for: Outcomes of Self-Expanding Versus Balloon-Expandable Transcatheter Aortic Valves in Patients With Reduced Left Ventricular Ejection Fraction: A Meta-Analysis of Observational Studies
Source: Struct Heart. 2026 Mar 30;10(6):100843. doi: 10.1016/j.shj.2026.100843 (PMC13200108; doi:10.1016/j.shj.2026.100843)
Supplement: Supplementary Material [file mmc1.docx]

These supplementary files for the manuscript titled “**Meta-analysis of Self-Expandable Versus Balloon-Expandable Valves in Aortic Stenosis with Reduced Ejection Fraction: A Call for Randomized Trials**”

Table of Contents

[Table S1: PRISMA Checklist 2](#_Toc222079557)

[Table S2: Detailed search strategy 5](#_Toc222079558)

[Table S3. Results of multivariate meta-analysis: effects of moderators on outcomes 7](#_Toc222079559)

[Table S4: Newcastle-Ottawa quality assessment scale of included studies in meta-analysis 9](#_Toc222079560)

[Table S5: Summary of the absolute rates in categorical outcomes 10](#_Toc222079561)

[Figure S1: One-year LVEF change (%) 11](#_Toc222079562)

[Figure S2: Aortic valve area (cmZ) 11](#_Toc222079563)

[Figure S3: Procedural mortality 11](#_Toc222079564)

[Figure S4: Thirty-day all-cause mortality 12](#_Toc222079565)

[Figure S5: Acute kidney injury 12](#_Toc222079566)

[Figure S6: Major/life-threatening bleeding 12](#_Toc222079567)

[Figure S7: Major vascular complications 12](#_Toc222079568)

[Figure S8: Minor vascular complications 13](#_Toc222079569)

# Table S1: PRISMA Checklist.

| **Section/Topic** | **Item #** | **Prisma 2020 Checklist item for manuscript** | **Location** |
| --- | --- | --- | --- |
| **TITLE** | | |  |
| Title | 1 | Identify the report as a systematic review. | 1 |
| **ABSTRACT** | | |  |
| Abstract | 2 | See the PRISMA 2020 for Abstracts checklist. | 1-2 |
| **INTRODUCTION** | | |  |
| Rationale | 3 | Describe the rationale for the review in the context of existing knowledge. | 1 |
| Objectives | 4 | Provide an explicit statement of the objective(s) or question(s) the review addresses. | 3 |
| **METHODS** | | |  |
| Eligibility criteria | 5 | Specify the inclusion and exclusion criteria for the review and how studies were grouped for the syntheses. | 3-4 |
| Information sources | 6 | Specify all databases, registers, websites, organisations, reference lists and other sources searched or consulted to identify studies. Specify the date when each source was last searched or consulted. | 4 |
| Search strategy | 7 | Present the full search strategies for all databases, registers and websites, including any filters and limits used. | 4 |
| Selection process | 8 | Specify the methods used to decide whether a study met the inclusion criteria of the review, including how many reviewers screened each record and each report retrieved, whether they worked independently, and if applicable, details of automation tools used in the process. | 4 |
| Data collection process | 9 | Specify the methods used to collect data from reports, including how many reviewers collected data from each report, whether they worked independently, any processes for obtaining or confirming data from study investigators, and if applicable, details of automation tools used in the process. | 5 |
| Data items | 10a | List and define all outcomes for which data were sought. Specify whether all results that were compatible with each outcome domain in each study were sought (e.g. for all measures, time points, analyses), and if not, the methods used to decide which results to collect. | 5 |
|  | 10b | List and define all other variables for which data were sought (e.g. participant and intervention characteristics, funding sources). Describe any assumptions made about any missing or unclear information. | 5 |
| Study risk of bias assessment | 11 | Specify the methods used to assess risk of bias in the included studies, including details of the tool(s) used, how many reviewers assessed each study and whether they worked independently, and if applicable, details of automation tools used in the process. | 4 |
| Effect measures | 12 | Specify for each outcome the effect measure(s) (e.g. risk ratio, mean difference) used in the synthesis or presentation of results. | 4-5 |
| Synthesis methods | 13a | Describe the processes used to decide which studies were eligible for each synthesis (e.g. tabulating the study intervention characteristics and comparing against the planned groups for each synthesis (item #5)). | 4 |
|  | 13b | Describe any methods required to prepare the data for presentation or synthesis, such as handling of missing summary statistics, or data conversions. | 5 |
|  | 13c | Describe any methods used to tabulate or visually display results of individual studies and syntheses. | 5 |
|  | 13d | Describe any methods used to synthesize results and provide a rationale for the choice(s). If meta-analysis was performed, describe the model(s), method(s) to identify the presence and extent of statistical heterogeneity, and software package(s) used. | 5 |
|  | 13e | Describe any methods used to explore possible causes of heterogeneity among study results (e.g. subgroup analysis, meta-regression). | 5 |
|  | 13f | Describe any sensitivity analyses conducted to assess robustness of the synthesized results. | 5 |
| Reporting bias assessment | 14 | Describe any methods used to assess risk of bias due to missing results in a synthesis (arising from reporting biases). | 5 |
| Assessment | 15 | Describe any Certainty methods used to assess certainty (or confidence) in the body of evidence for an outcome. | 5 |
| **RESULTS** | | |  |
| Study selection | 16a | Describe the results of the search and selection process, from the number of records identified in the search to the number of studies included in the review, ideally using a flow diagram. | 5 |
|  | 16b | Cite studies that might appear to meet the inclusion criteria, but which were excluded, and explain why they were excluded. | 5 |
| Study characteristics | 17 | Cite each included study and present its characteristics. | 5 |
| Risk of bias in studies | 18 | Present assessments of risk of bias for each included study. | 5 |
| Results of individual studies | 19 | For all outcomes, present, for each study: (a) summary statistics for each group (where appropriate) and (b) an effect estimate and its precision (e.g. confidence/credible interval), ideally using structured tables or plots. | 5-6 |
| Results of syntheses | 20a | For each synthesis, briefly summarise the characteristics and risk of bias among contributing studies. | 5-6 |
|  | 20b | Present results of all statistical syntheses conducted. If meta-analysis was done, present for each the summary estimate and its precision (e.g. confidence/credible interval) and measures of statistical heterogeneity. If comparing groups, describe the direction of the effect. | 5-6 |
|  | 20c | Present results of all investigations of possible causes of heterogeneity among study results. | 5-6 |
|  | 20d | Present results of all sensitivity analyses conducted to assess the robustness of the synthesized results. | 5-6 |
| Reporting biases | 21 | Present assessments of risk of bias due to missing results (arising from reporting biases) for each synthesis assessed. | 5-6 |
| Certainty of evidence | 22 | Present assessments of certainty (or confidence) in the body of evidence for each outcome assessed. | 5-6 |
| **DISCUSSION** | | |  |
| Discussion | 23a | Provide a general interpretation of the results in the context of other evidence. | 6-7 |
|  | 23b | Discuss any limitations of the evidence included in the review. | 6-7 |
|  | 23c | Discuss any limitations of the review processes used. | 7-8 |
|  | 23d | Discuss implications of the results for practice, policy, and future research. | 7-8 |
| **OTHER INFORMATION** | | |  |
| Registration and protocol | 24a | Provide registration information for the review, including register name and registration number, or state that the review was not registered. | 4 |
|  | 24b | Indicate where the review protocol can be accessed, or state that a protocol was not prepared. | 4 |
|  | 24c | Describe and explain any amendments to information provided at registration or in the protocol. | N/A |
| Support | 25 | Describe sources of financial or non-financial support for the review, and the role of the funders or sponsors in the review. | 8-9 |
| Competing interests | 26 | Declare any competing interests of review authors. | 8-9 |
| Availability of data, code and other materials | 27 | Report which of the following are publicly available and where they can be found: template data collection forms; data extracted from included studies; data used for all analyses; analytic code; any other materials used in the review. | 9 |

# Table S2: Detailed search strategy.

| Database | Search term | Result |
| --- | --- | --- |
| Pubmed | ("Transcatheter Aortic Valve Replacement"[Mesh] OR "Aortic Valve Stenosis"[Mesh] OR TAVR OR TAVI OR Aortic Stenosis OR Aortic Supravalvular Stenosis OR aortic valve intervention OR transcatheter heart valve OR Percutaneous aortic valve replacement) AND (low ejection fraction OR LVEF OR reduced ejection fraction OR REF OR impaired ejection fraction OR Left Ventricular Dysfunction* OR LV Dysfunction* OR Left Ventricular Diastolic Dysfunction) AND (Self-expanding transcatheter heart valve OR SEV OR SEP OR CoreValve OR Balloon-expandable transcatheter heart valves OR BEV OR BEP OR "Evolut*" OR Portico OR Sapien* ) | 1,039 |
| WOS | (Transcatheter Aortic Valve Replacement OR Aortic Valve Stenosis OR TAVR OR TAVI OR Aortic Stenosis OR Aortic Supravalvular Stenosis OR Percutaneous aortic valve replacement) AND (low ejection fraction OR LVEF OR reduced ejection fraction OR Left Ventricular Dysfunction) AND (Self expanding transcatheter heart valve OR SEV OR SEP OR Balloon expandable transcatheter heart valves OR BEV OR BEP OR (Evolut) OR Sapien) | 208 |
| Scopus | (search in: title, Abstracts and key words)  ((Transcatheter aortic valve replacement) OR (TAVR) OR (Transcatheter Aortic Valve Implantation) OR (TAVI) OR (Aortic Valve Stenoses) OR (Stenoses, Aortic Valve) OR (Stenosis, Aortic Valve) OR (Valve Stenoses, Aortic) OR (Valve Stenosis, Aortic) OR (Aortic Stenosis) OR (Stenoses, Aortic) OR (Stenosis, Aortic) OR (Aortic Stenosis, Calcific) OR (Aortic Supravalvular Stenosis) OR (Aortic Supravalvular Stenoses) OR (aortic valve intervention) OR (transcatheter heart valve) OR (Percutaneous aortic valve replacement)) AND ((low ejection fraction) OR (LVEF) OR (reduced ejection fraction) OR (REF) OR (impaired ejection fraction) OR (Left Ventricular Dysfunction*) OR (LV Dysfunction*) OR (Left Ventricular Diastolic Dysfunction) OR (Diastolic Dysfunction, LV) OR (Dysfunction, LV Diastolic) OR (LV Diastolic Dysfunction*)) AND ((Self-expanding transcatheter heart valve) OR (SEV) OR (SEP) OR (CoreValve) OR (Balloon-expandable transcatheter heart valves) OR (BEV) OR (BEP) OR (Evolut) OR (Sapien)) | 620 |
| Cochrane | (Transcatheter Aortic Valve Replacement OR Aortic Valve Stenosis OR TAVR OR TAVI OR Aortic Stenosis OR Aortic Supravalvular Stenosis OR aortic valve intervention OR transcatheter heart valve OR Percutaneous aortic valve replacement) AND (low ejection fraction OR LVEF OR reduced ejection fraction OR REF OR impaired ejection fraction OR Left Ventricular Dysfunction OR LV Dysfunction OR Left Ventricular Diastolic Dysfunction) AND (Self-expanding transcatheter heart valve OR SEV OR SEP OR CoreValve OR Balloon-expandable transcatheter heart valves OR BEV OR BEP OR "Evolut" OR Sapien ) | 33 |
| Embase | (Transcatheter Aortic Valve Replacement OR Aortic Valve Stenosis OR TAVR OR TAVI OR Aortic Stenosis OR Aortic Supravalvular Stenosis OR aortic valve intervention OR transcatheter heart valve OR Percutaneous aortic valve replacement) AND (low ejection fraction OR LVEF OR reduced ejection fraction OR REF OR impaired ejection fraction OR Left Ventricular Dysfunction OR LV Dysfunction OR Left Ventricular Diastolic Dysfunction) AND (Self-expanding transcatheter heart valve OR SEV OR SEP OR CoreValve OR Balloon-expandable transcatheter heart valves OR BEV OR BEP OR "Evolut" OR Portico OR Jena Valve OR Sapien ) | 1765 |

#

# Table S3: Results of multivariate meta-analysis: effects of moderators on outcomes.

| **Covariate** | **Effect (95%CI)** | ***p*** | **Residual Heterogeneity *p*** | **Test of Moderators** |
| --- | --- | --- | --- | --- |
| **1-month LVEF improvement (%)** | | | | |
| Valve type (SEV vs BEV) | 0.321 (0.122, 0.521) | **< 0.01** | 0.989 | 0.11 |
| Female % | -0.04 (-0.081, -0.002) | **0.04** | 0.979 |  |
| Baseline LVEF | -0.135 (-0.376, 0.107) | 0.275 | 0.987 |  |
| Baseline transaortic gradient | -0.009 (-0.087, 0.068) | 0.817 | 0.989 |  |
| **1-year LVEF improvement (%)** | | | | |
| Valve type (SEV vs BEV) | -0.164 (-0.388, 0.06) | 0.15 | 1 | < 0.01 |
| Female % | 0.053 (0.025, 0.081) | **< 0.01** | 1 |  |
| **Mean aortic gradient (mmHg)** | | | | |
| Valve type (SEV vs BEV) | -0.683 (-0.887, -0.48) | **< 0.01** | 0.979 | < 0.01 |
| Female % | 0.019 (-0.021, 0.06) | 0.357 | 0.987 |  |
| Baseline LVEF | -0.267 (-0.511, -0.022) | **0.0329** | 0.989 |  |
| Baseline transaortic gradient | 0.072 (-0.006, 0.151) | 0.0705 | 0.979 |  |
| **Aortic valve area (cm²)** | | | | |
| Valve type (SEV vs BEV) | 0.068 (-0.13, 0.266) | 0.5 | 0.987 | 0.174 |
| Female % | 0.001 (-0.039, 0.04) | 0.979 | 0.989 |  |
| Baseline LVEF | 0.104 (-0.137, 0.346) | 0.397 | 0.979 |  |
| Baseline transaortic gradient | 0.039 (-0.039, 0.116) | 0.329 | 0.987 |  |
| **Procedural mortality** | | | | |
| Valve type (SEV vs BEV) | 1.291 (0.189, 8.824) | 0.795 | 0.839 | 0.379 |
| Female % | 0.947 (0.791, 1.133) | 0.551 | 0.839 |  |
| Baseline LVEF | 0.783 (0.476, 1.289) | 0.337 | 0.839 |  |
| History of pacemaker implantation | 0.943 (0.76, 1.171) | 0.595 | 0.839 |  |
| **30-day all-cause mortality** | | | | |
| Valve type (SEV vs BEV) | 0.557 (0.142, 2.187) | 0.402 | 0.362 | < 0.01 |
| Female % | 1.056 (0.963, 1.158) | 0.244 | 0.362 |  |
| Baseline LVEF | 1.011 (0.66, 1.546) | 0.962 | 0.362 |  |
| History of pacemaker implantation | 1.082 (1.007, 1.164) | **0.0323** | 0.362 |  |
| **1-year all-cause mortality** | | | | |
| Valve type (SEV vs BEV) | 1.13 (0.694, 1.838) | 0.623 | 0.495 | < 0.01 |
| Female % | 1.019 (0.978, 1.062) | 0.358 | 0.495 |  |
| Baseline LVEF | 1.222 (0.994, 1.504) | 0.0576 | 0.495 |  |
| History of pacemaker implantation | 1.058 (1.015, 1.103) | **< 0.01** | 0.495 |  |
| **Stroke** | | | | |
| Valve type (SEV vs BEV) | 1.761 (0.315, 9.828) | 0.519 | 0.618 | 0.085 |
| Female % | 0.966 (0.842, 1.108) | 0.623 | 0.618 |  |
| Baseline LVEF | 1.411 (0.855, 2.329) | 0.178 | 0.618 |  |
| History of pacemaker implantation | 1.13 (1.011, 1.263) | **0.0315** | 0.618 |  |
| **Acute kidney injury** | | | | |
| Valve type (SEV vs BEV) | 0.534 (0.053, 5.378) | 0.595 | 0.188 | 0.531 |
| Female % | 1.096 (0.87, 1.38) | 0.436 | 0.188 |  |
| Baseline LVEF | 1.075 (0.639, 1.809) | 0.785 | 0.188 |  |
| History of pacemaker implantation | 0.92 (0.53, 1.598) | 0.768 | 0.188 |  |
| **Major/life-threatening bleeding** | | | | |
| Valve type (SEV vs BEV) | 0.696 (0.126, 3.84) | 0.677 | 0.198 | 0.324 |
| Female % | 1.005 (0.855, 1.181) | 0.954 | 0.198 |  |
| Baseline LVEF | 1.277 (0.747, 2.185) | 0.372 | 0.198 |  |
| History of pacemaker implantation | 1.026 (0.935, 1.126) | 0.587 | 0.198 |  |
| **Major vascular complications** | | | | |
| Valve type (SEV vs BEV) | 1.621 (0.109, 24.062) | 0.726 | 0.0962 | 0.026 |
| Female % | 1.999 (1.159, 3.449) | **0.0128** | 0.0962 |  |
| Baseline LVEF | 1.702 (0.91, 3.183) | 0.096 | 0.0962 |  |
| History of pacemaker implantation | 0.294 (0.098, 0.877) | **0.0281** | 0.0962 |  |
| **Minor vascular complications** | | | | |
| Valve type (SEV vs BEV) | 2.086 (0.505, 8.613) | 0.31 | 0.64 | 0.116 |
| Female % | 1.356 (0.952, 1.933) | 0.0919 | 0.64 |  |
| Baseline LVEF | 1.159 (0.668, 2.011) | 0.599 | 0.64 |  |
| History of pacemaker implantation | 0.501 (0.241, 1.041) | 0.0639 | 0.64 |  |
| **New pacemaker implantation** | | | | |
| Valve type (SEV vs BEV) | 1.056 (0.703, 1.586) | 0.791 | 0.149 | 0.344 |
| Female % | 1.021 (0.982, 1.062) | 0.294 | 0.149 |  |
| Baseline LVEF | 1.022 (0.852, 1.226) | 0.818 | 0.149 |  |
| History of pacemaker implantation | 1.008 (0.951, 1.068) | 0.787 | 0.149 |  |
| **Paravalvular leak (>mild)** | | | | |
| Valve type (SEV vs BEV) | 2.521 (1.457, 4.364) | **< 0.01** | 0.235 | < 0.01 |
| Female % | 1.004 (0.938, 1.075) | 0.898 | 0.235 |  |
| Baseline LVEF | 1.214 (0.9, 1.638) | 0.205 | 0.235 |  |
| History of pacemaker implantation | 1.006 (0.923, 1.097) | 0.891 | 0.235 |  |

| Table S4. Newcastle-Ottawa quality assessment scale of included studies in meta-analysis. | | | | | | | | | |
| --- | --- | --- | --- | --- | --- | --- | --- | --- | --- |
|  | **Selection** | | | |  | **Outcome** | | |  |
| First author, Year | Represent-ativeness | Selection of comparison cohort | Ascertain-ment | Endpoint not present at start | **Comparability (cofounding)** | Assessment of outcome | Follow up duration | Adequacy follow up | **Total score** |
| El-Chilali 2020 | * | * | * | * |  | * | * | * | 7 |
| Matta 2024 | * | * | * | * |  | * | * | * | 7 |
| Mustafa 2022 | * | * | * | * | * | * | * | * | 8 |
| Nakase 2025 | * | * | * | * | ** | * | * | * | 9 |
| Giordano 2023 | * | * | * | * | ** | * | * | * | 9 |
| Notes. The Newcastle-Ottawa scale uses a star system (0 to 9) to evaluate included studies on 3 domains, selection, comparability, and outcomes. Star (*) item presents Maximum 1 star (*) for selection and outcome components and 2 stars (**) for comparability components. Higher scores represent higher study quality | | | | | | | | | |

#

# Table S5: Summary of the absolute rates in categorical outcomes.

| **Outcome** | **Studies** | **Participants (sev vs bev)** | **% of patients experienced outcome (SEV)** | **% of patients experienced outcome (BEV)** |
| --- | --- | --- | --- | --- |
| Procedural mortality | 2 | 992 vs 691 | 1.2 | 1.4 |
| 30-day all-cause mortality | 3 | 329 vs 415 | 6.4 | 4.6 |
| 1-year all-cause mortality | 4 | 975 vs 692 | 13.8 | 12.4 |
| Stroke | 5 | 1041 vs 783 | 2.8 | 1.3 |
| Acute kidney injury | 3 | 261 vs 372 | 5.7 | 6.7 |
| Major/life-threatening bleeding | 4 | 895 vs 594 | 7.9 | 7.9 |
| Major vascular complications | 4 | 907 vs 649 | 3.6 | 3.4 |
| Minor vascular complications | 4 | 907 vs 649 | 4.9 | 5.5 |
| New pacemaker implantation | 5 | 1041 vs 783 | 11.7 | 12.6 |
| Paravalvular Leak (>mild) | 5 | 1041 vs 783 | 13.7 | 7.8 |

# Figure S1: One-year LVEF change (%)


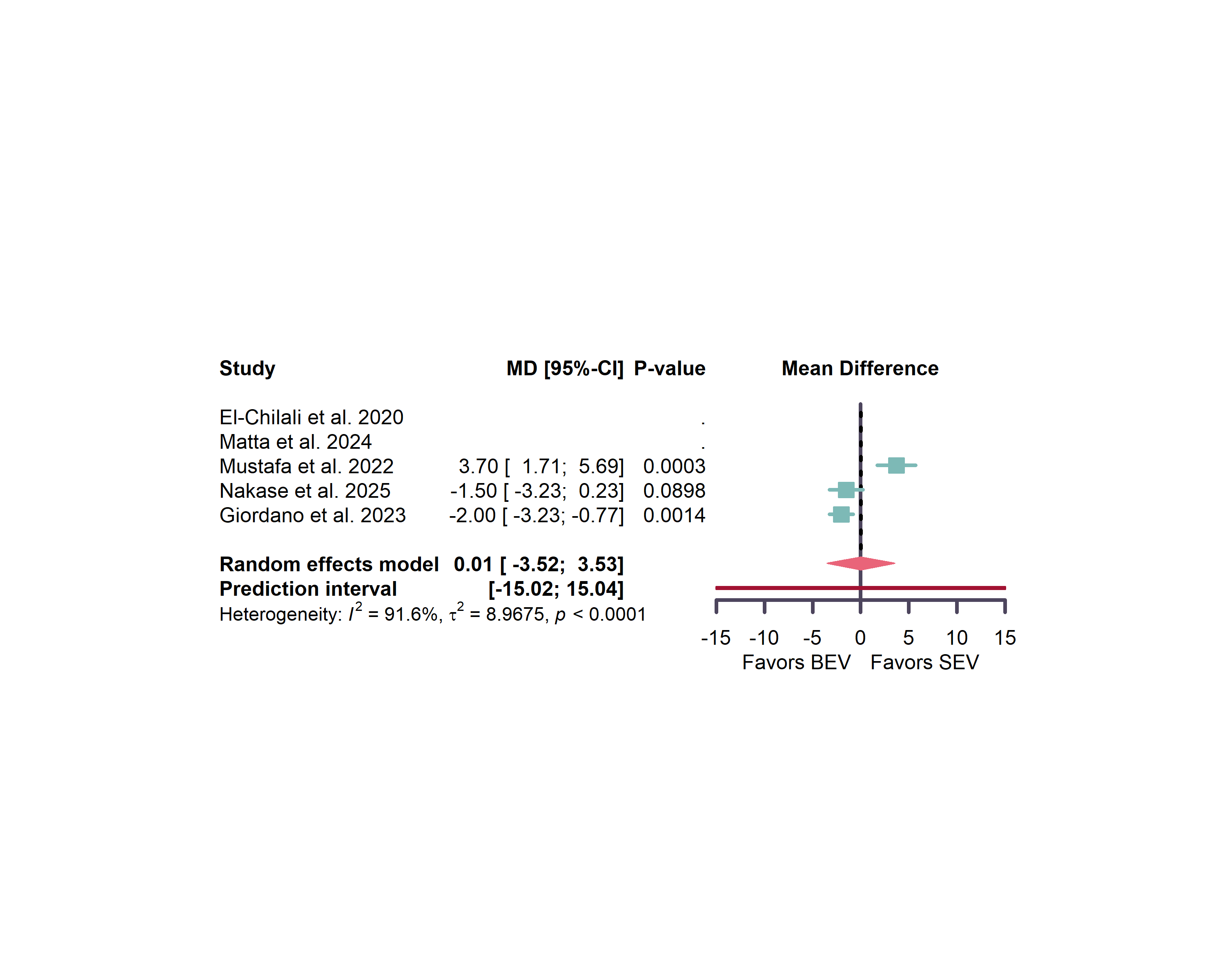


# Figure S2: Aortic valve area (cmZ)

**
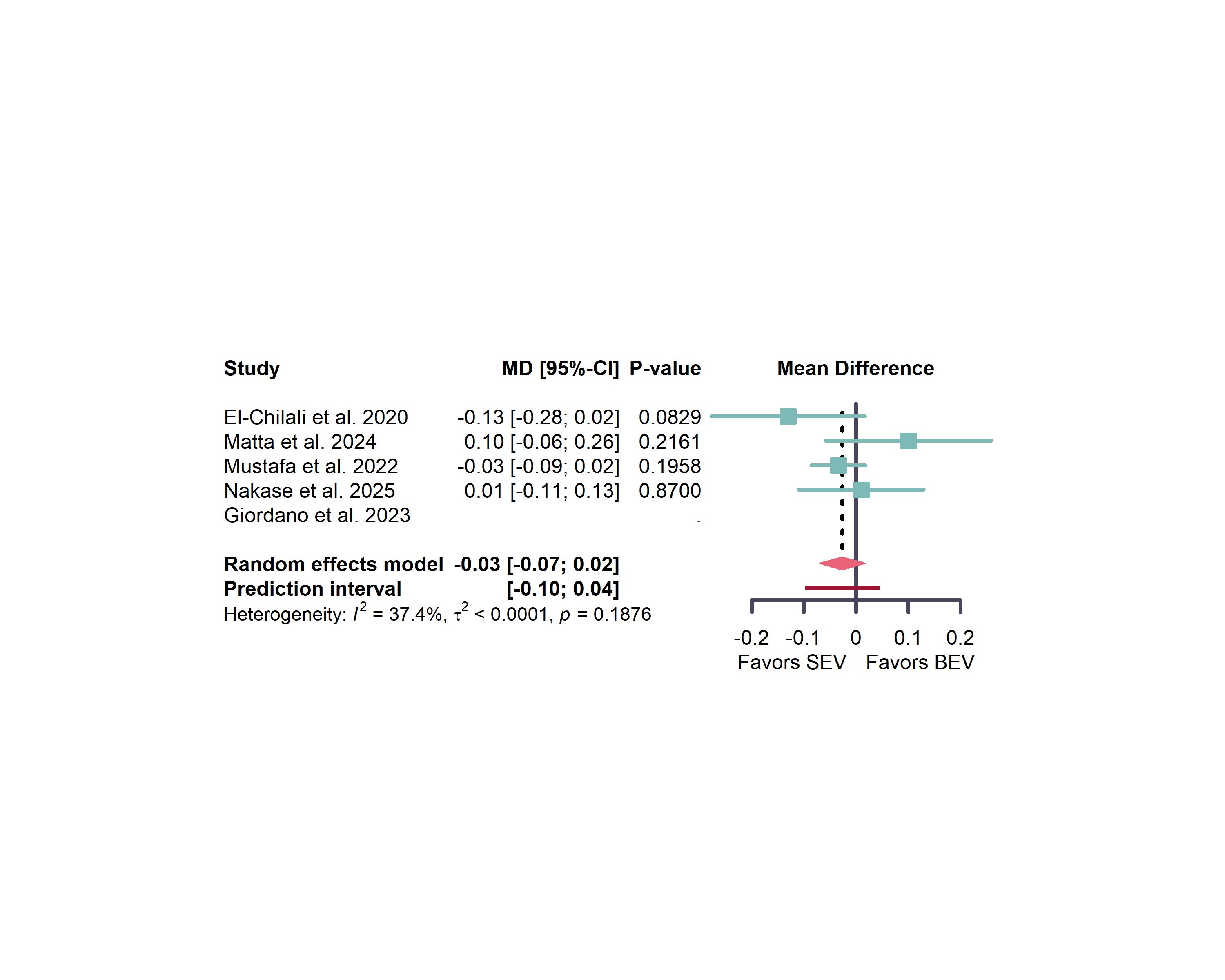
**

# Figure S3: Procedural mortality

**
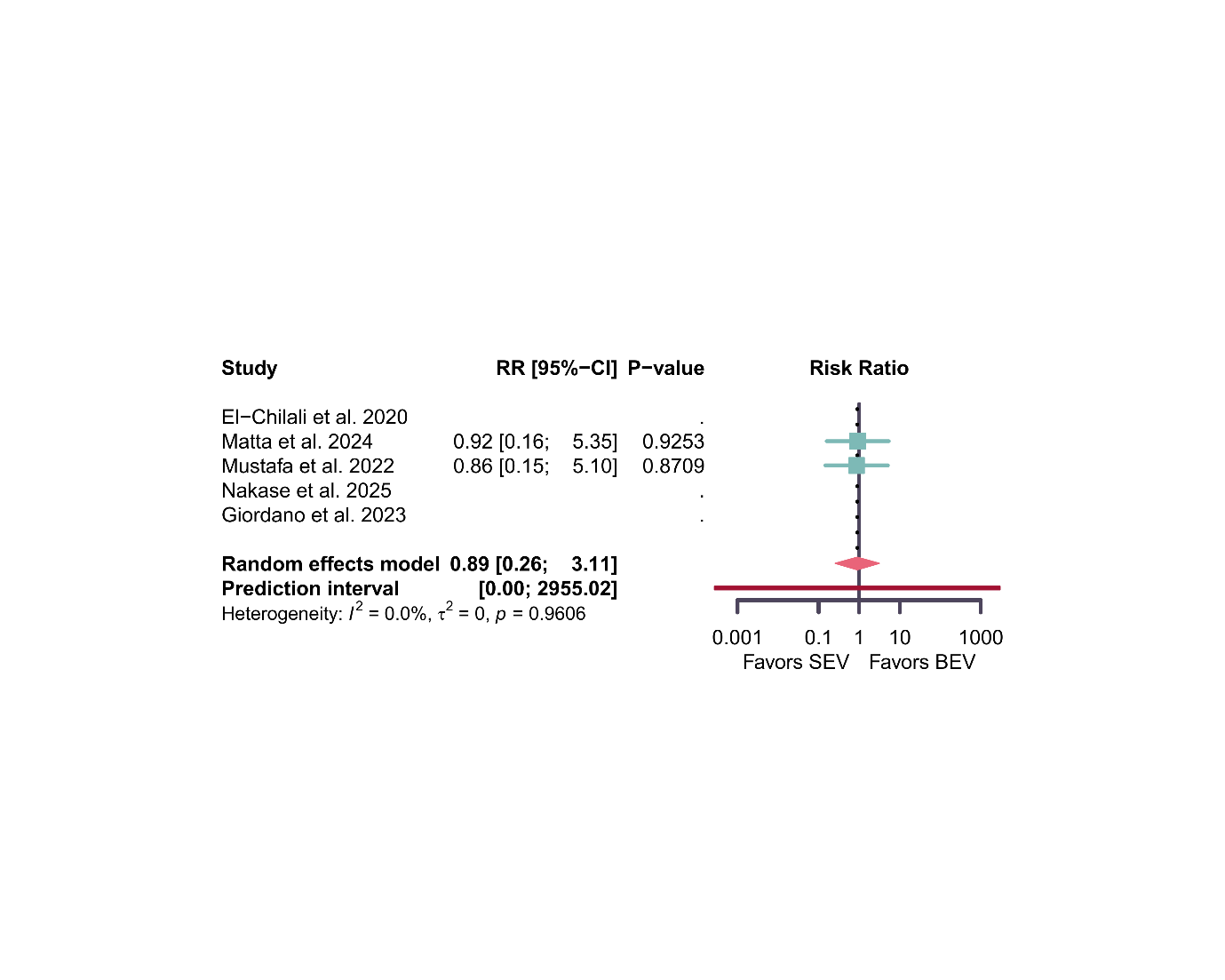
**

# Figure S4: Thirty-day all-cause mortality

**
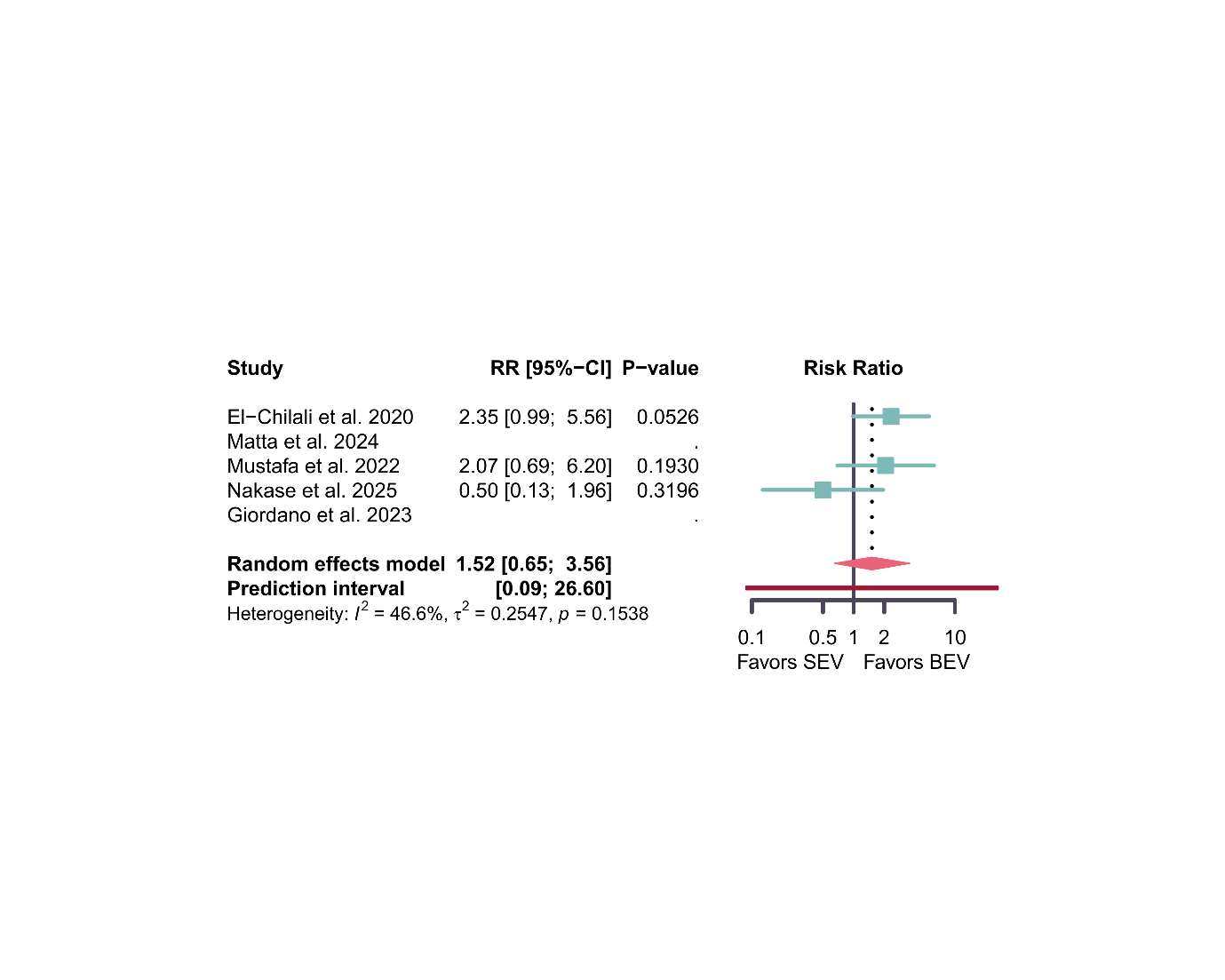
**

# Figure S5: Acute kidney injury

**
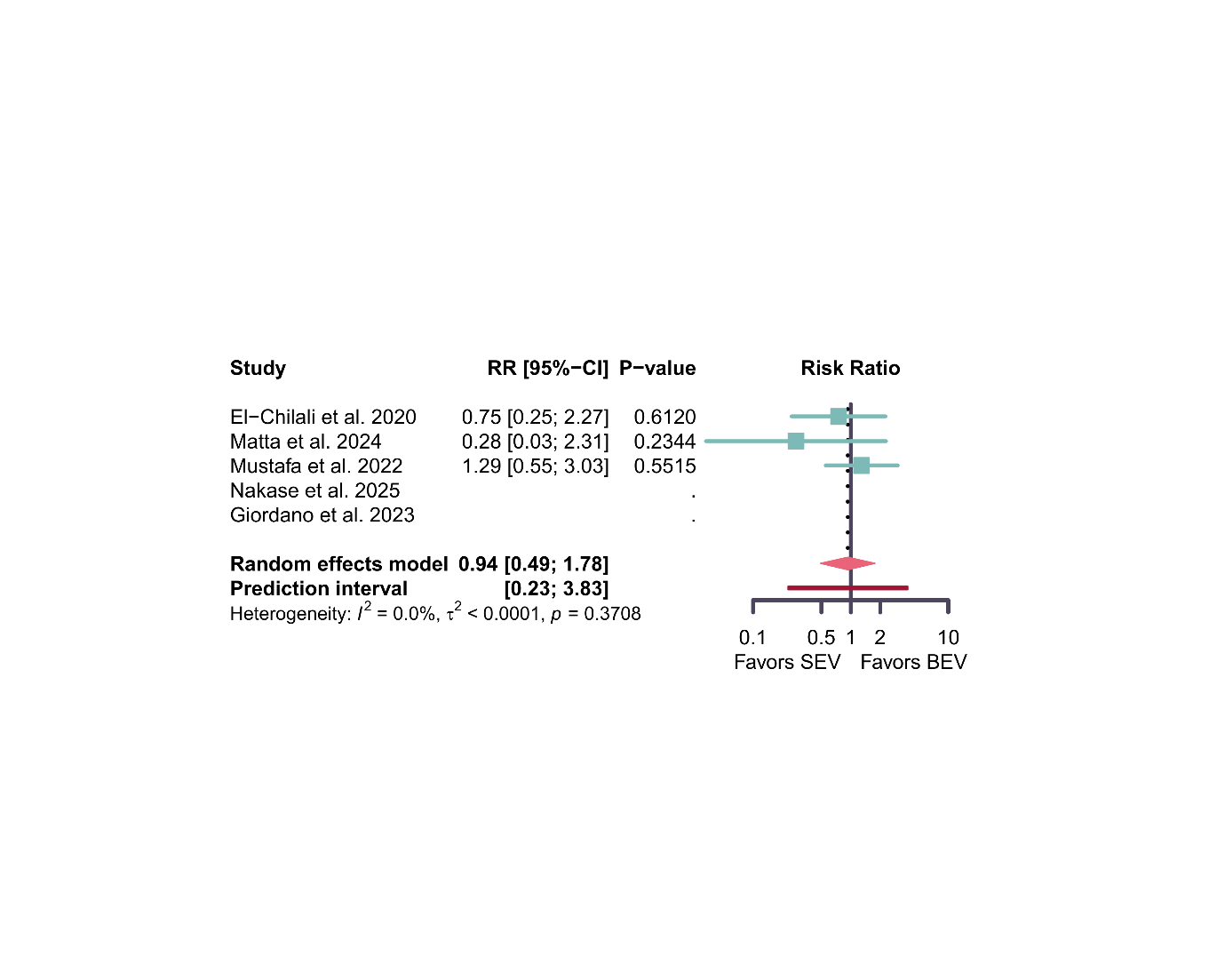
**

# Figure S6: Major/life-threatening bleeding

**
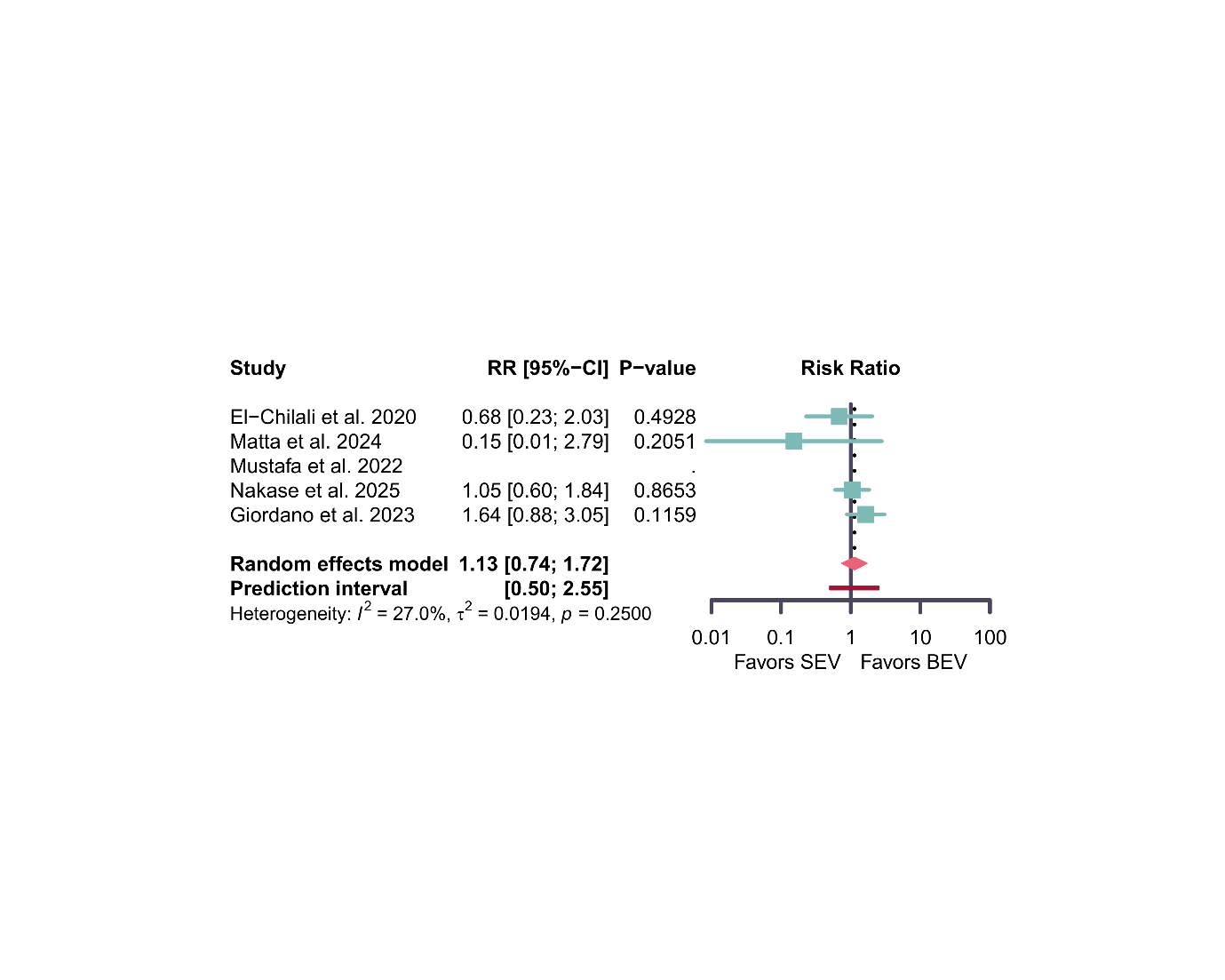
**

# Figure S7: Major vascular complications

**
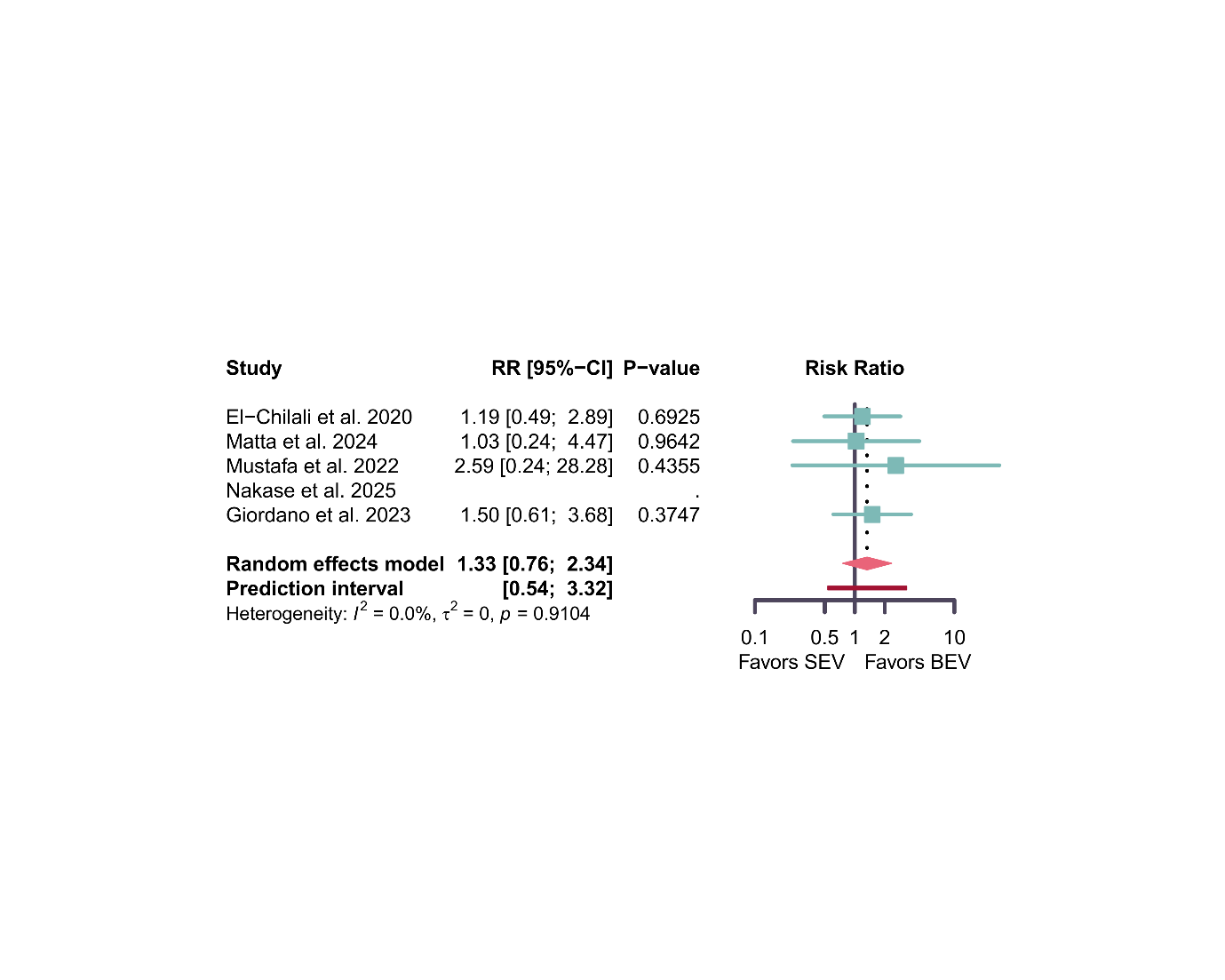
**

# Figure S8: Minor vascular complications

**
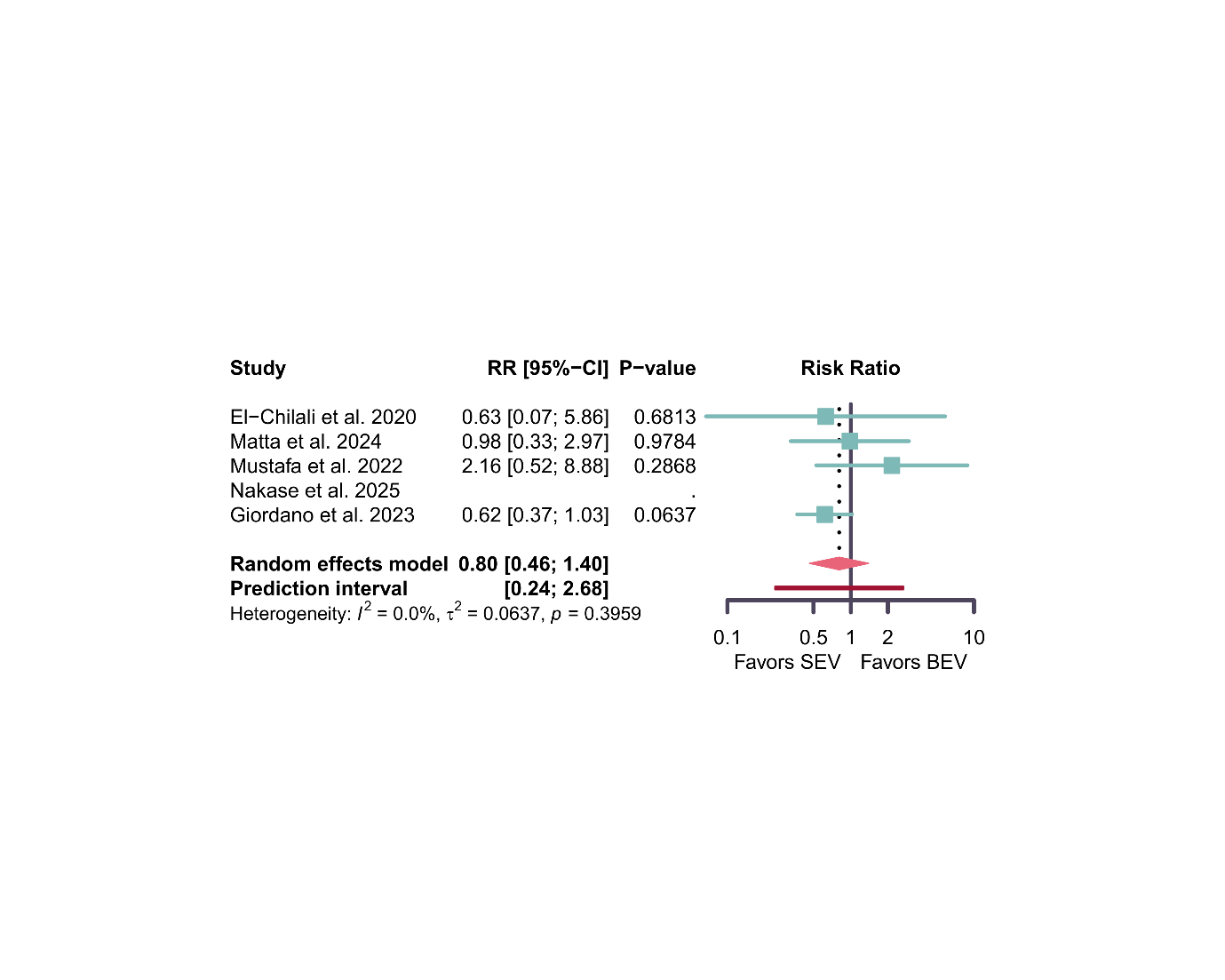
**
